# Supplementary material for: Distributed representations of prediction error signals across the cortical hierarchy are synergistic
Source: Nat Commun. 2024 May 10;15:3941. doi: 10.1038/s41467-024-48329-7 (PMC11087548; doi:10.1038/s41467-024-48329-7)
Supplement: Supplementary file 1 — Supplementary Information [file 41467_2024_48329_MOESM1_ESM.pdf]

Supplementary Materials for

Distributed representations of prediction error signals across the cortical hierarchy are synergistic

Frank Gelens, Juho Äijälä, Louis Roberts, Misako Komatsu, Cem Uran, Michael A. Jensen, Kai J. Miller, Robin A.A. Ince, Max Garagnani, Martin Vinck, Andres Canales-Johnson.

* Correspondence to: afc37@cam.ac.uk; martin.vinck@esi-frankfurt.de

This PDF file includes:

Supplementary Figures S1 to S16

Supplementary Methods

Supplementary Figures

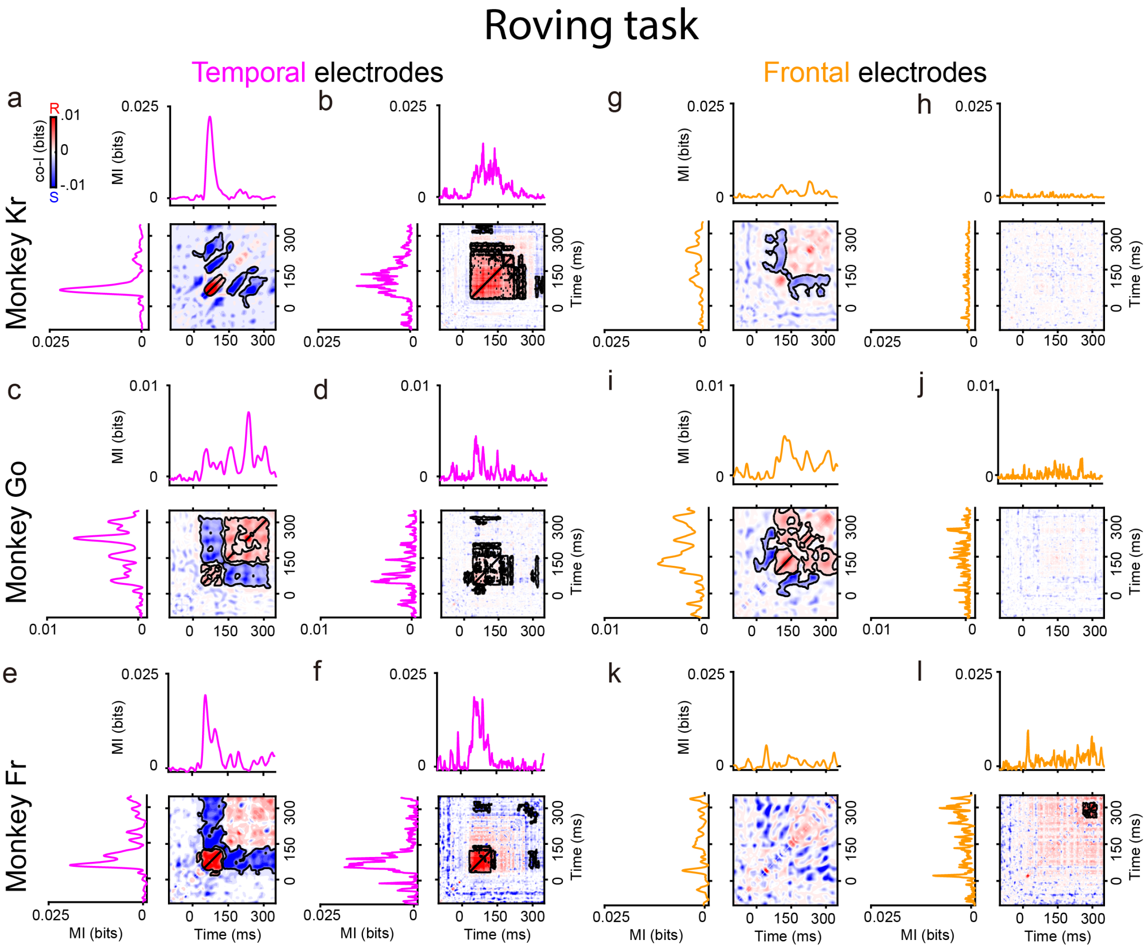

Figure S1: Synergy and redundancy within ERP and within BB signals in temporal and frontal electrodes with the highest MI for the roving task for monkeys Kr, Go and Fr. Co-information within auditory **(a, c, e)**, and frontal **(g, i, k)** electrodes in the ERP signal. Co-information within auditory **(b, d, f)**, and frontal **(h, j, l)** electrodes in the BB signal. MI (solid traces) between standard and deviant trials for temporal (pink color) and frontal (orange color) electrodes. Co-I was computed between each pair of electrodes and across time points between -100 to 350 ms after tone presentation. Significant temporal clusters after a permutation test (see Methods) are depicted in black contours. Source data are provided as a Source Data file.

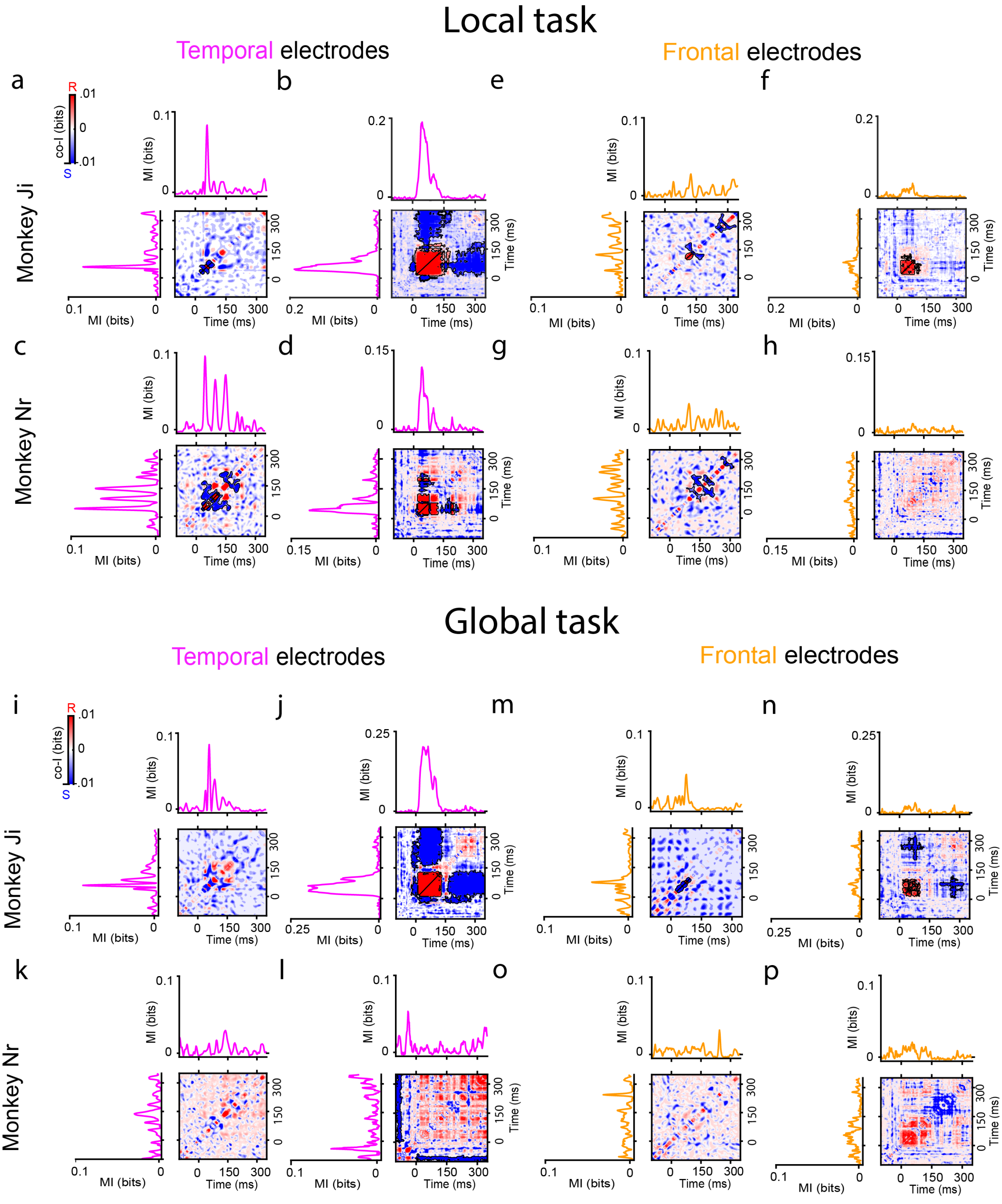

Figure S2: Synergy and redundancy within ERP and within BB signals in temporal and frontal electrodes with the highest MI for the local and global deviants of the local-global task Co-information within auditory **(a, c)**, and frontal **(e, g)** electrodes in the ERP signal for the local task. Co-information within auditory **(b, d)**, and frontal **(f, h)** electrodes in the BB signal for the local task. Co-information within auditory **(i, k)**, and frontal **(m, o)** electrodes in the ERP signal for the global task. Co-information within auditory **(j, l)**, and frontal **(n, p)** electrodes in the BB signal for the global task. MI (solid traces) between standard and deviant trials for temporal (pink color) and frontal (orange color) electrodes. Co-I was computed between each pair of electrodes and across time points between -100 to 350 ms after tone presentation. Significant temporal clusters after a permutation test (see Methods) are depicted in black contours. Source data are provided as a Source Data file.

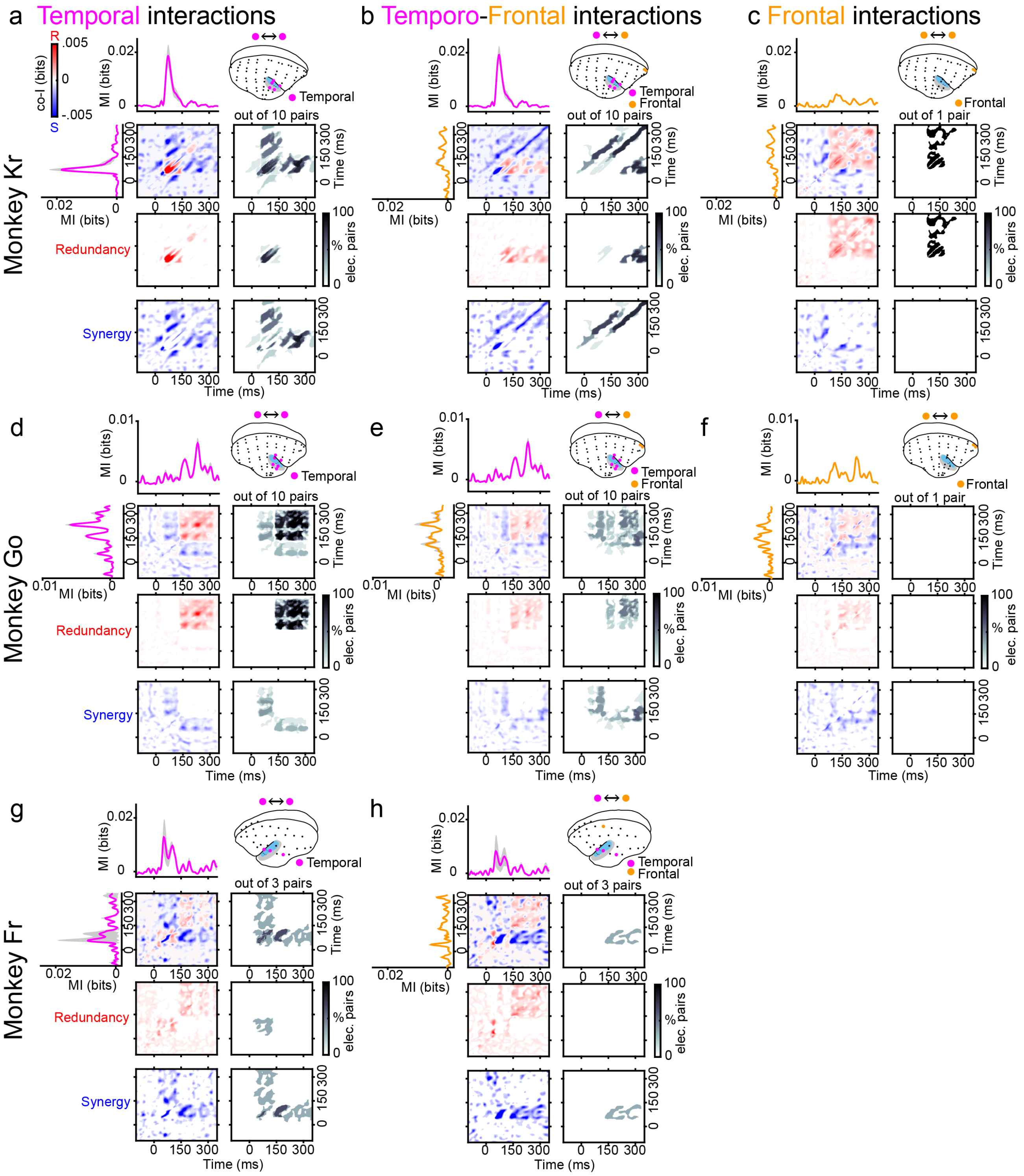

Figure S3: Synergy and redundancy between ERP signals and across cortical areas in marmosets Kr, Go and Fr. Co-information revealed synergistic and redundant PE patterns across temporal **(a, d, g)**, temporo-frontal **(b, e, h)**, and frontal **(c, f)** electrodes. MI (solid traces) between standard and deviant trials for temporal (pink color) and frontal (orange color) electrodes. Error bars represent standard error of the mean (S.E.M) across electrodes. Co-I was computed between each pair of electrodes and across time points between -100 to 350 ms after tone presentation. The average of the corresponding electrode pairs per (i.e. temporal, temporo-frontal, and frontal) is shown for the complete co-I values (red and blue panel), for positive co-I values (redundancy only; red panel), and negative co-I values (synergy only; blue panel). Source data are provided as a Source Data file. Panels **a-h** are adapted from Komatsu, M., Takaura, K. & Fujii, N. Mismatch negativity in common marmosets: Whole-cortical recordings with multi-channel electrocorticograms. Sci Rep 5, 15006 (2015). https://doi.org/10.1038/srep15006, under a CC-BY license: https://creativecommons.org/licenses/by/4.0/.

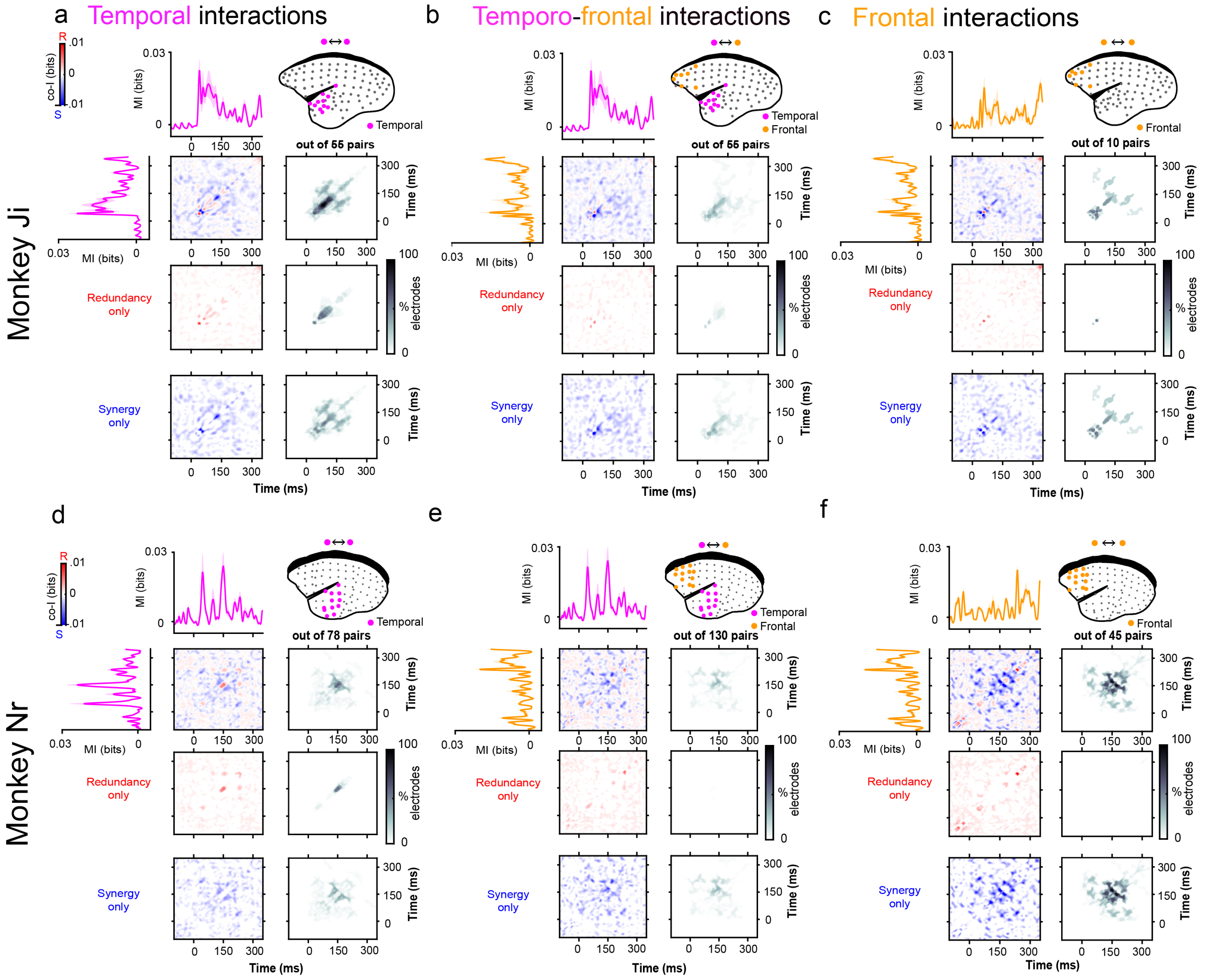

Figure S4: Synergy and redundancy between ERP signals and across cortical areas for marmosets Ji and Nr for the local deviant of the Local-Global task. Co-information revealed synergistic and redundant PE patterns across temporal **(a, d)**, temporo-frontal **(b, e)**, and frontal **(c, f)** electrodes. MI (solid traces) between standard and deviant trials for temporal (pink color) and frontal (orange color) electrodes. Error bars represent standard error of the mean (S.E.M) across electrodes. Co-I was computed between each pair of electrodes and across time points between -100 to 350 ms after tone presentation. The average of the corresponding electrode pairs per (i.e. temporal, temporo-frontal, and frontal) is shown for the complete co-I values (red and blue panel), for positive co-I values (redundancy only; red panel), and negative co-I values (synergy only; blue panel). Source data are provided as a Source Data file. Panels **a**-**f** are adapted from Yuwei Jiang, et al. (2022) Constructing the hierarchy of predictive auditory sequences in the marmoset brain eLife 11:e74653. https://doi.org/10.7554/eLife.74653, under a CC-BY license: https://creativecommons.org/licenses/by/4.0/.

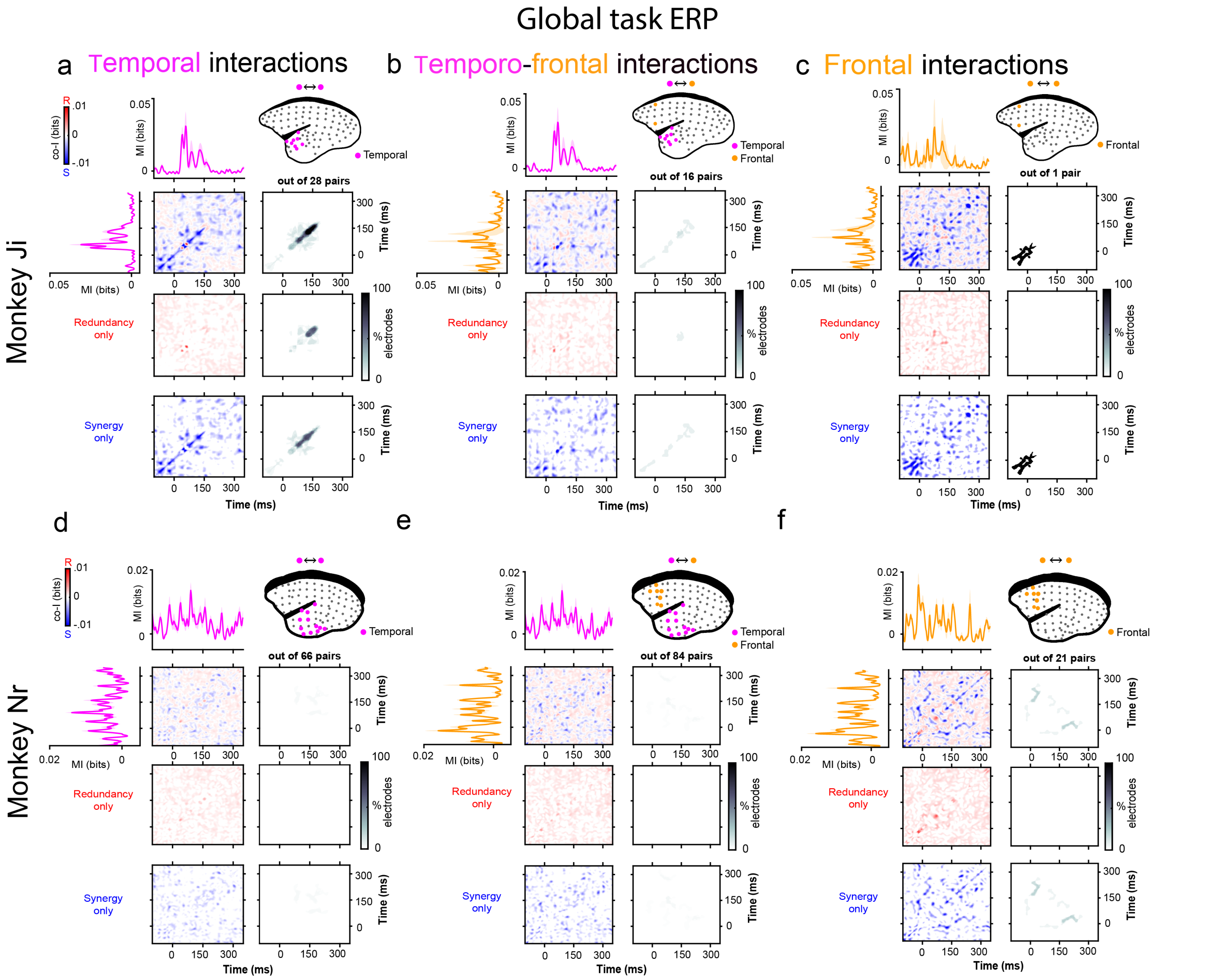

Figure S5: Synergy and redundancy between ERP signals and across cortical areas for marmosets Ji and Nr for the Global deviant of the Local-Global task. Co-information revealed synergistic and redundant PE patterns across temporal **(a, d)**, temporo-frontal (**b, e**), and frontal **(c, f)** electrodes mostly for marmoset Ji, while Nr only showed relatively weak synergetic patterns between frontal electrodes. MI (solid traces) between standard and deviant trials for temporal (pink color) and frontal (orange color) electrodes. Error bars represent standard error of the mean (S.E.M) across electrodes. Co-I was computed between each pair of electrodes and across time points between -100 to 350 ms after tone presentation. The average of the corresponding electrode pairs per (i.e. temporal, temporo-frontal, and frontal) is shown for the complete co-I values (red and blue panel), for positive co-I values (redundancy only; red panel), and negative co-I values (synergy only; blue panel). Source data are provided as a Source Data file. Panels **a-f** are adapted from Yuwei Jiang, et al. (2022) Constructing the hierarchy of predictive auditory sequences in the marmoset brain eLife 11:e74653. https://doi.org/10.7554/eLife.74653, under a CC-BY license: https://creativecommons.org/licenses/by/4.0/.

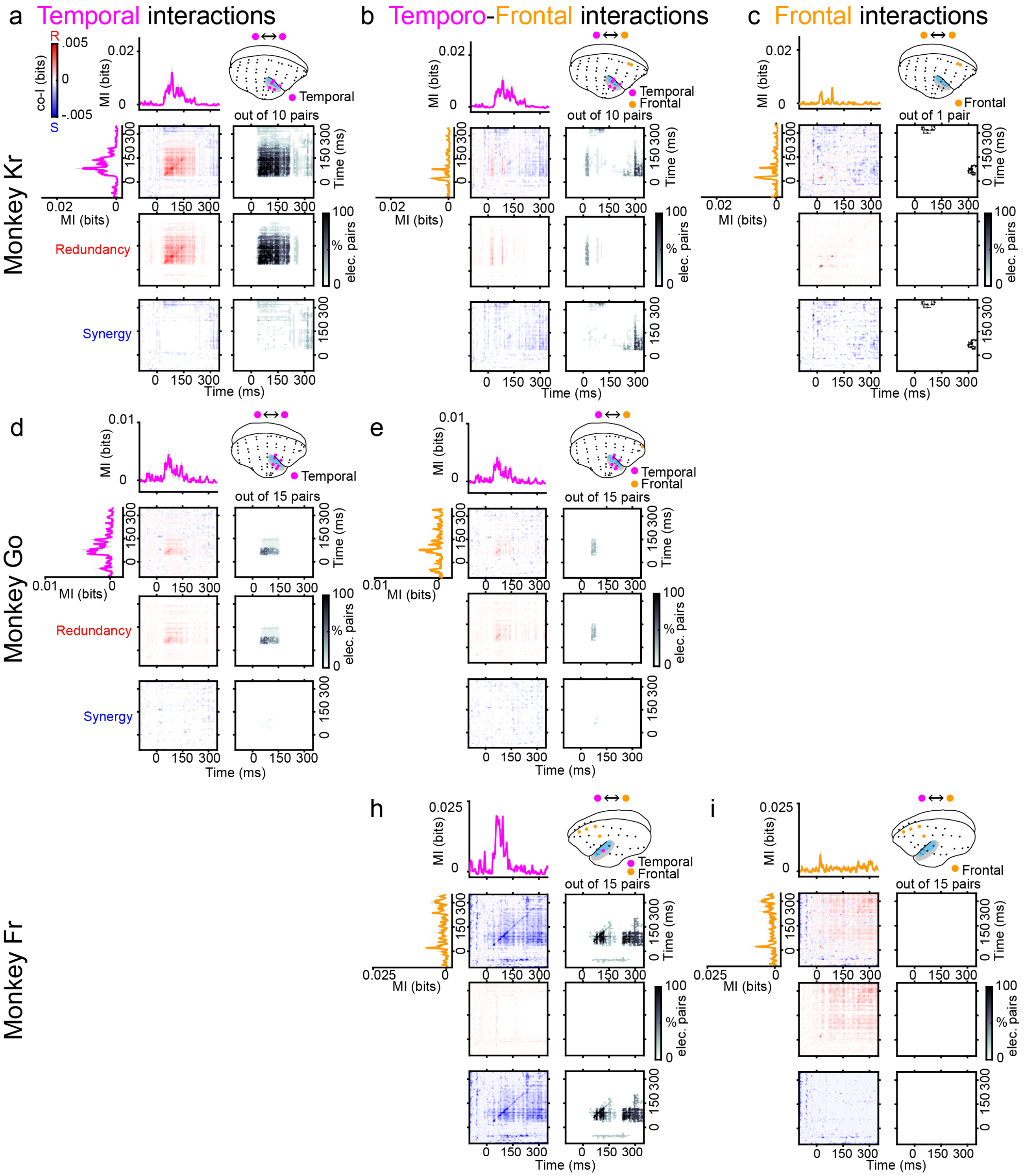

Figure S6: Synergy and redundancy between BB signals and across cortical areas in marmosets Kr, Go and Fr in the Roving task. Co-information revealed synergistic and redundant PE patterns across temporal **(a, d)**, temporo-frontal **(b, e, h)**, and frontal **(c, i)** electrodes. MI (solid traces) between standard and deviant trials for temporal (pink color) and frontal (orange color) electrodes. Error bars represent standard error of the mean (S.E.M) across electrodes. Co-I was computed between each pair of electrodes and across time points between -100 to 350 ms after tone presentation. The average of the corresponding electrode pairs per (i.e. temporal, temporo-frontal, and frontal) is shown for the complete co-I values (red and blue panel), for positive co-I values (redundancy only; red panel), and negative co-I values (synergy only; blue panel). Source data are provided as a Source Data file. Panels **a**-**i** are adapted from Komatsu, M., Takaura, K. & Fujii, N. Mismatch negativity in common marmosets: Whole-cortical recordings with multi-channel electrocorticograms. Sci Rep 5, 15006 (2015). https://doi.org/10.1038/srep15006, under a CC-BY license: https://creativecommons.org/licenses/by/4.0/.

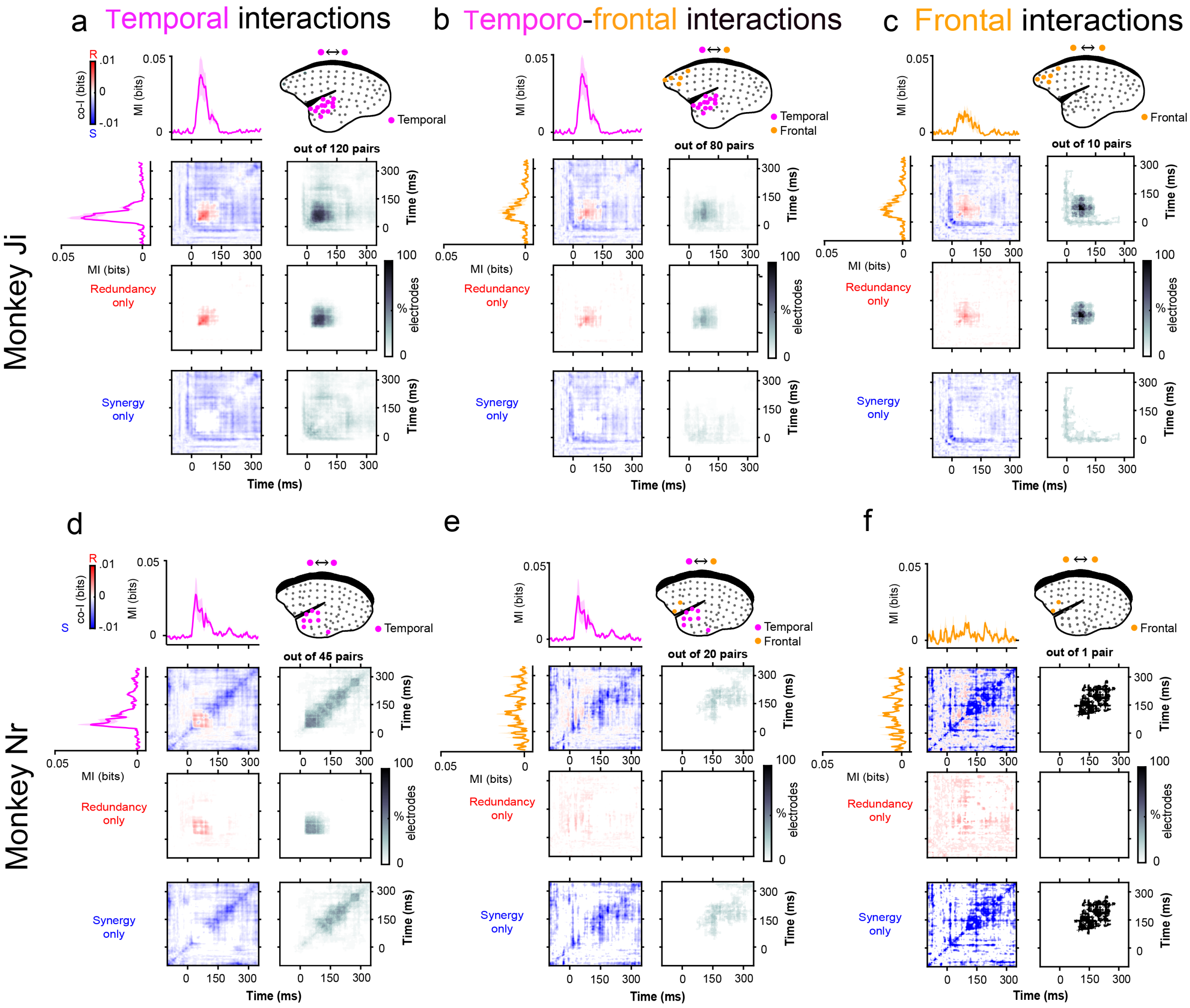

Figure S7: Synergy and redundancy between BB signals and across cortical areas for marmosets Ji and Nr for the local deviant of the Local-Global task. Co-information revealed synergistic and redundant PE patterns across temporal **(a, d)**, temporo-frontal **(b, e)**, and frontal **(c, f)** electrodes. MI (solid traces) between standard and deviant trials for temporal (pink color) and frontal (orange color) electrodes. Error bars represent standard error of the mean (S.E.M) across electrodes. Co-I was computed between each pair of electrodes and across time points between -100 to 350 ms after tone presentation. The average of the corresponding electrode pairs per (i.e. temporal, temporo-frontal, and frontal) is shown for the complete co-I values (red and blue panel), for positive co-I values (redundancy only; red panel), and negative co-I values (synergy only; blue panel). Source data are provided as a Source Data file. Panels **a-f** are adapted from Yuwei Jiang, et al. (2022) Constructing the hierarchy of predictive auditory sequences in the marmoset brain eLife 11:e74653. https://doi.org/10.7554/eLife.74653, under a CC-BY license: https://creativecommons.org/licenses/by/4.0/".

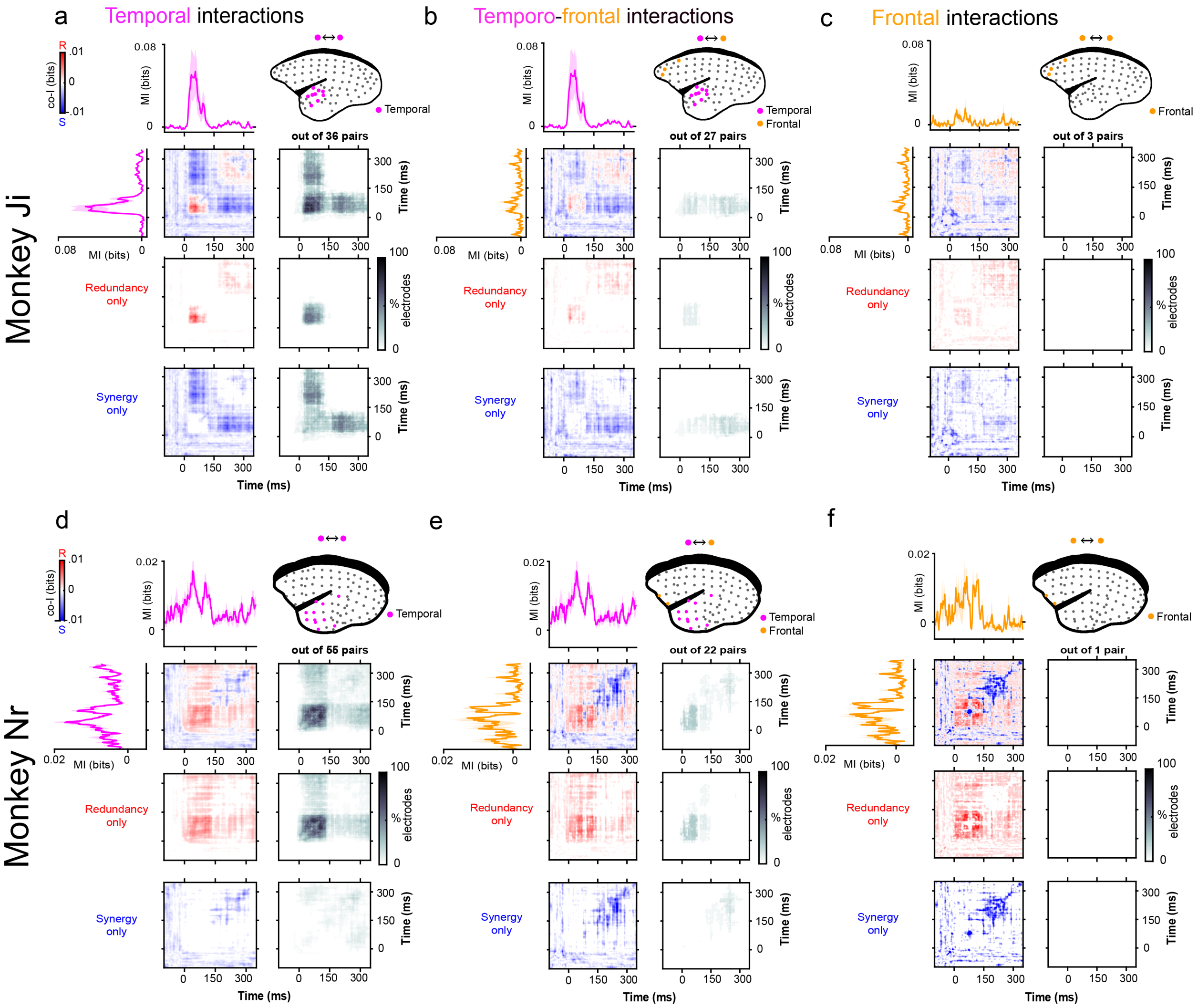

Figure S8: Synergy and redundancy between ERP signals and across cortical areas in marmosets Ji and Nr for the global deviant of the Local-Global task. Co-information revealed synergistic and redundant PE patterns across temporal **(a, d)**, temporo-frontal **(b, e)**, and frontal **(c, f)** electrodes. MI (solid traces) between standard and deviant trials for temporal (pink color) and frontal (orange color) electrodes. Error bars represent standard error of the mean (S.E.M) across electrodes. Co-I was computed between each pair of electrodes and across time points between -100 to 350 ms after tone presentation. The average of the corresponding electrode pairs per (i.e. temporal, temporo-frontal, and frontal) is shown for the complete co-I values (red and blue panel), for positive co-I values (redundancy only; red panel), and negative co-I values (synergy only; blue panel). Source data are provided as a Source Data file. Panels **a**-**f** are adapted from Yuwei Jiang, et al. (2022) Constructing the hierarchy of predictive auditory sequences in the marmoset brain eLife 11:e74653. https://doi.org/10.7554/eLife.74653, under a CC-BY license: https://creativecommons.org/licenses/by/4.0/.

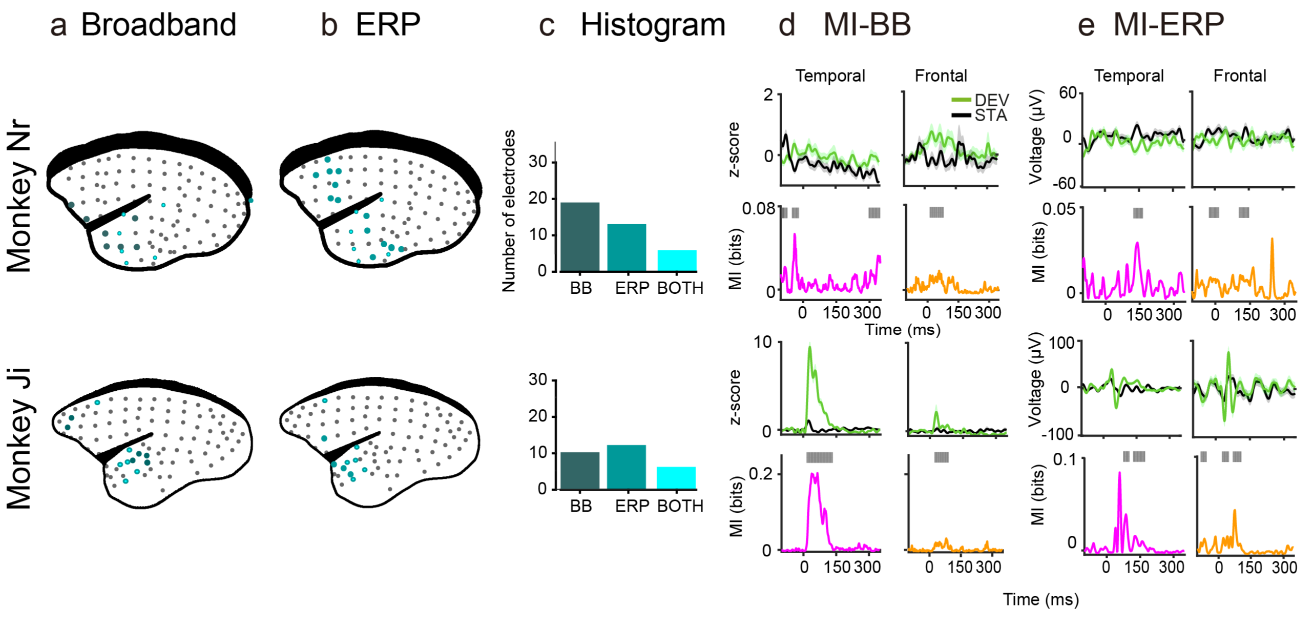

Figure S9: Broadband and ERP markers of PE across the monkey brain for monkeys Ji and Nr for the global task. Electrode locations for marmoset Nr (96 electrodes; upper panel) and Ji (96 electrodes; lower panel). Electrodes showing significant PE effect after computing MI between standard and deviant trials for the **(a)** Broadband (dark green circles) and **(b)** ERP (light green circles) markers of auditory prediction error in both monkeys. Electrodes showing significant MI for both markers are depicted in cyan. **(c)** Histogram of electrodes showing significant MI between tones for BB (left), ERP (middle), and both markers (right) for each animal. **(d)** Electrodes with the highest MI in the temporal and frontal cortex showing the BB signal for deviant and standard tones. Error bars represent standard error of the mean (S.E.M) across trials. Deviant tone (green) and standard tone (black), and the corresponding MI values in bits (effect size of the difference) for the temporal (pink trace) and frontal (orange trace) electrodes. Significant time points after a permutation test are shown as grey bars over the MI plots. **(e)** Electrodes with the highest MI in the temporal and frontal cortex showing the ERP signal for deviant and standard tones. Color codes are the same as in **c**. Source data are provided as a Source Data file. Panels **a-f** are adapted from Yuwei Jiang, et al. (2022) Constructing the hierarchy of predictive auditory sequences in the marmoset brain eLife 11:e74653. https://doi.org/10.7554/eLife.74653, under a CC-BY license: https://creativecommons.org/licenses/by/4.0/.

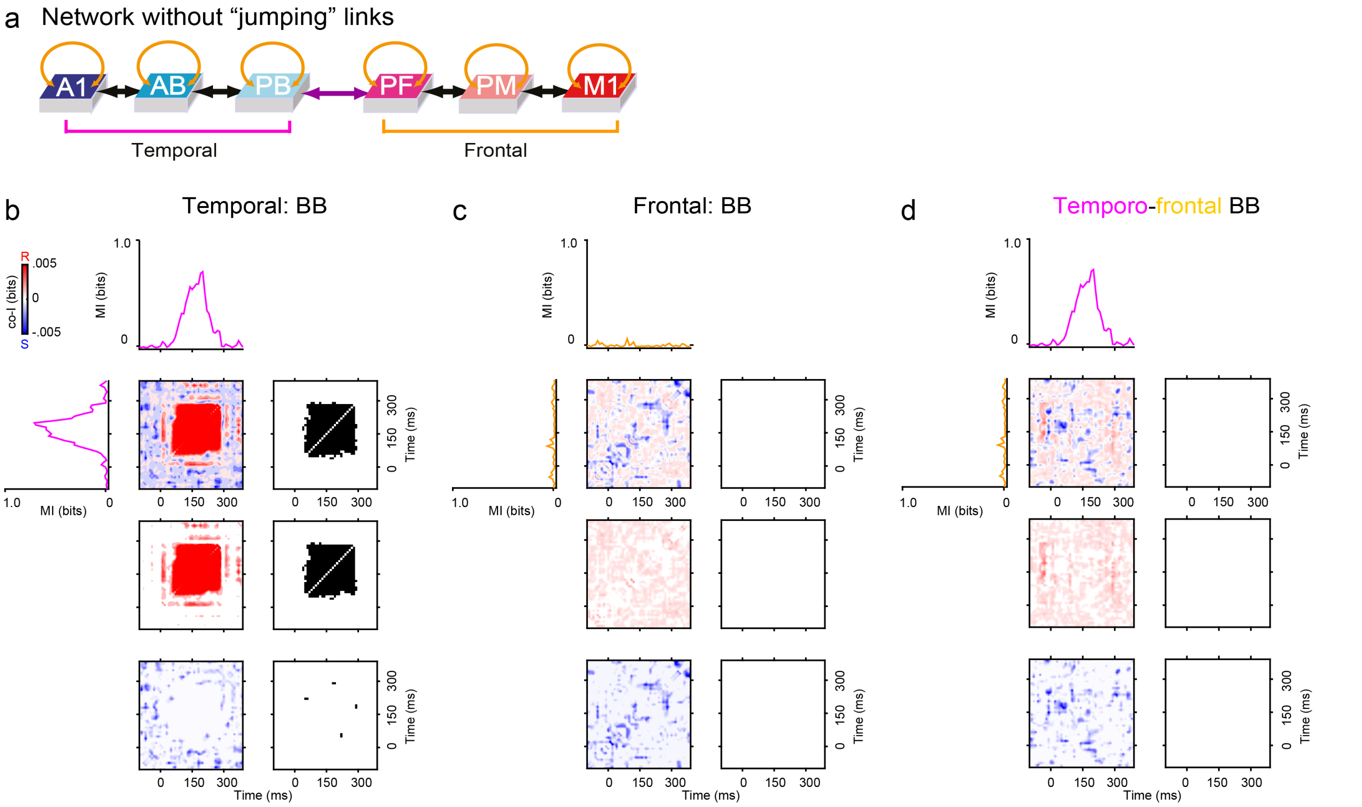

Figure S10: Results of network simulations. (**a**) Model architecture: fully connected (FC) with "serial" connectivity structure (i.e., only next-neighbour between-area links). Connections included both feedforward and feedback (black and green arrows) and recurrent (golden arrows) links, but, unlike in previous simulations, no higher-order "jumping" links (simulating cortico-cortical projections between non-adjacent areas A1-PB, AB-PF, PB-PM, and PB-M1). (**b, c, d**) Results obtained by stimulating the network of Panel **a** using a simulated Roving Task paradigm (as in Experiment 1). MI (solid traces) between standard and deviant trials are plotted for the three temporal (A1, AB, PB: pink curves) and three frontal (PF, PM, M1: orange curves) areas' simulated responses. Co-information analyses were performed between the simulated temporal and frontal areas' signals. Temporal co-I was computed from the simulated firing rates across time points between -100 to 350 ms after stimulus onset. The average of the corresponding electrodes for the simulated responses is shown for the complete co-I chart (red and blue panel), for positive co-I values (redundancy only; red panel), and negative co-I values (synergy only; blue panel). The grey-scale panels show significant clusters of co-I for areas with the highest MI. Note the almost entire absence of synergistic (or redundant) information between temporal and frontal regions (see panel **d**).

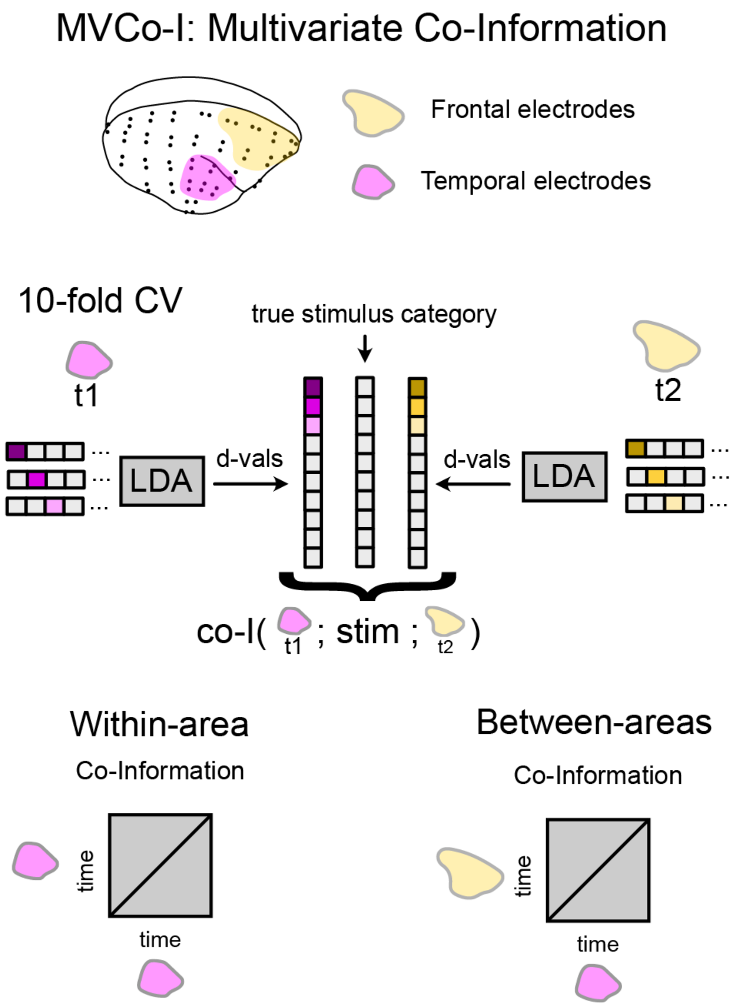

Figure S11: Schematic of the MVCo-I method. To calculate co-information between multivariate responses we use 10-fold cross-validation (CV) with Linear Discriminant Analysis (LDA). For each of the 10 hold-out folds, we train an LDA classifier to discriminate the stimulus category of a trial (i.e. deviant vs standard tone). We then compute the classifier decision values (d-vals; i.e. the linear combination of the learned pattern weights and the raw data) on each trial for the hold-out fold. We then concatenate the CV d-vals from all folds. We have used LDA as a cross-validated supervised dimensionality reduction method to obtain a one-dimensional representation of the region's activity at that time point, which is maximally discriminative between conditions. We can then compute the co-information between the ground-truth stimulus class of each trial, and the CV d-vals from two different classifiers (i.e. either from the same region at different time points, within-area coI, or from different regions, between-areas co-I). This calculation is the same as for channel-wise analysis (see Methods). Panels **a**-**i** are adapted from Komatsu, M., Takaura, K. & Fujii, N. Mismatch negativity in common marmosets: Whole-cortical recordings with multi-channel electrocorticograms. Sci Rep 5, 15006 (2015). https://doi.org/10.1038/srep15006, under a CC-BY license: https://creativecommons.org/licenses/by/4.0/.

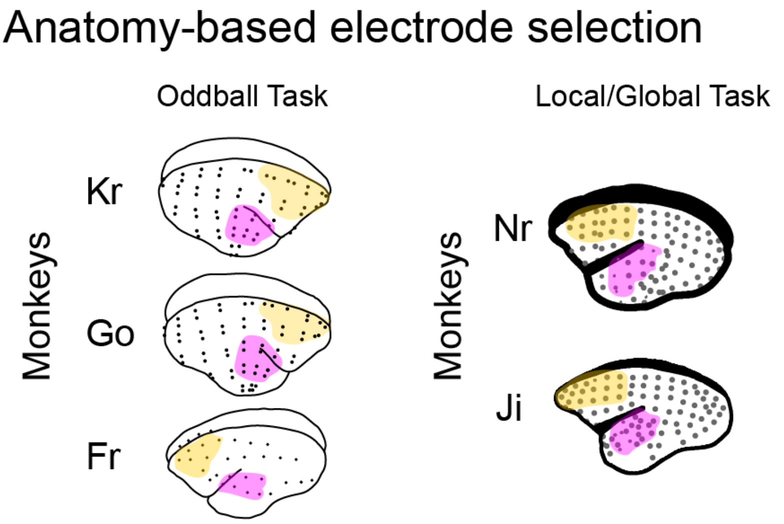

Figure S12: Anatomy-based electrode selection for the MVCo-I Method per marmoset. Roving Oddball Task (3 marmosets: Kr, Go and Fr) and Local/Global Task (2 marmosets: Ji and Nr). Brain layouts are adapted from Yuwei Jiang, et al. (2022) Constructing the hierarchy of predictive auditory sequences in the marmoset brain eLife 11:e74653. https://doi.org/10.7554/eLife.74653, under a CC-BY license: https://creativecommons.org/licenses/by/4.0/.

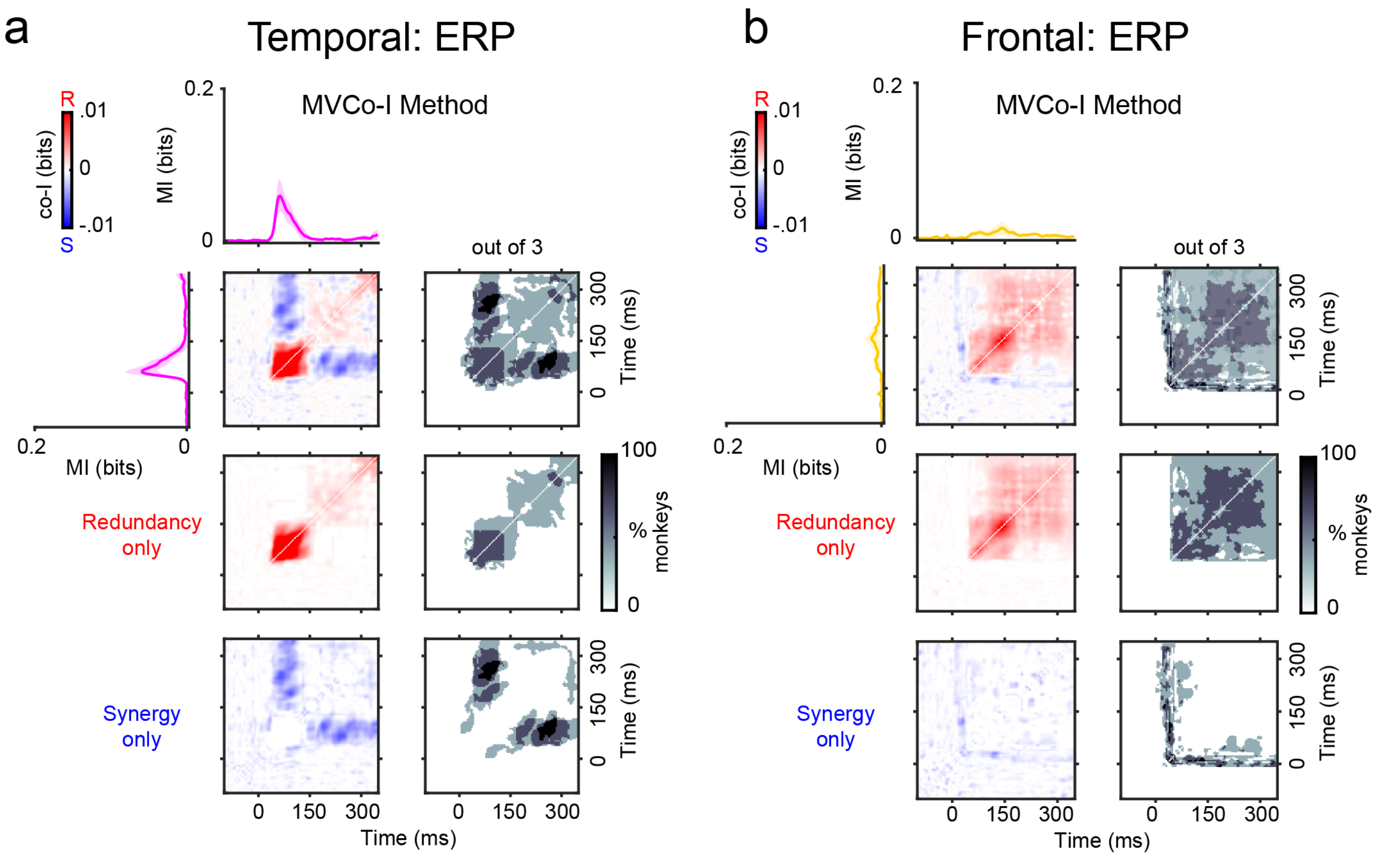

Figure S13: Temporal synergy and redundancy within ERP signals in the auditory and frontal electrodes using the MVCo-I Method (Experiment 1: Roving Oddball Task). MVCo-I revealed synergistic and redundant temporal patterns within Temporal ERP (Panel **a**) and Frontal ERP (Panel **b**) signals in the auditory cortex. MI (solid traces) between standard and deviant trials for auditory (pink color) and frontal (orange color) responses averaged across the three monkeys. The corresponding electrodes used for the MVCo-I method are depicted in Fig. 1b. Error bars represent standard error of the mean (S.E.M). Temporal co-I was computed within the corresponding signal (ERP) across time points between -100 to 350 ms after tone presentation. The average of the corresponding electrodes across monkeys is shown for the complete co-I chart (red and blue plots); for positive co-I values (redundancy only; red panel); and negative co-I values (synergy only; blue plot).

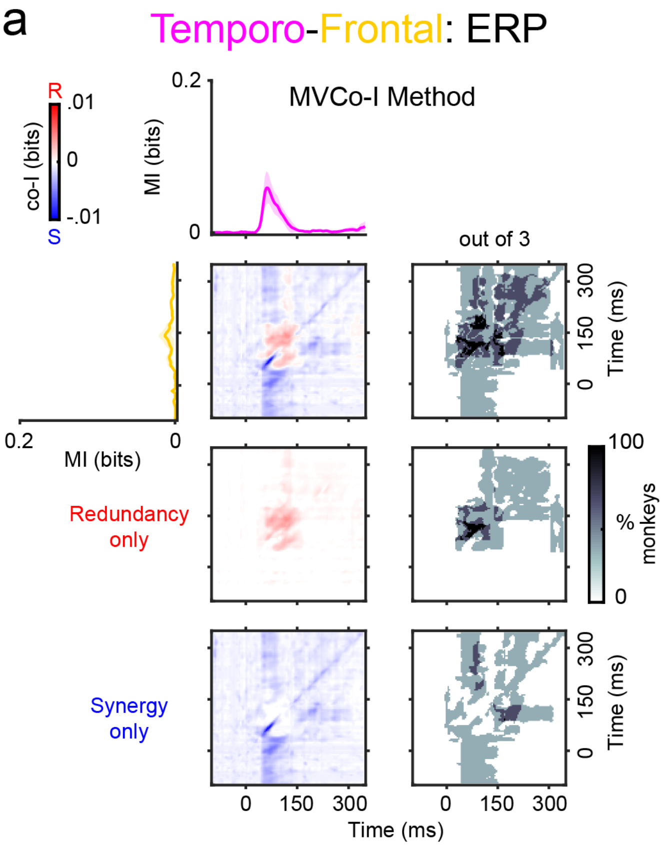

Figure S14: Spatio-temporal synergy and redundancy between auditory and frontal ERP signals using the MVCo-I Method (Experiment 1: Roving Oddball Task). (**a**) MVCo-I revealed synergistic and redundant temporal patterns between temporal and frontal signals. MI (solid traces) between standard and deviant trials for auditory (pink color) and frontal (orange color) responses averaged across the three monkeys. The corresponding electrodes used for the MVCo-I method are depicted in Fig. 1b. Error bars represent standard error of the mean (S.E.M). Temporal co-I was computed within the corresponding signal (ERP) across time points between -100 to 350 ms after tone presentation. The average of the corresponding electrodes across monkeys is shown for the complete co-I chart (red and blue plots); for positive co-I values (redundancy only; red panel); and negative co-I values (synergy only; blue plot).

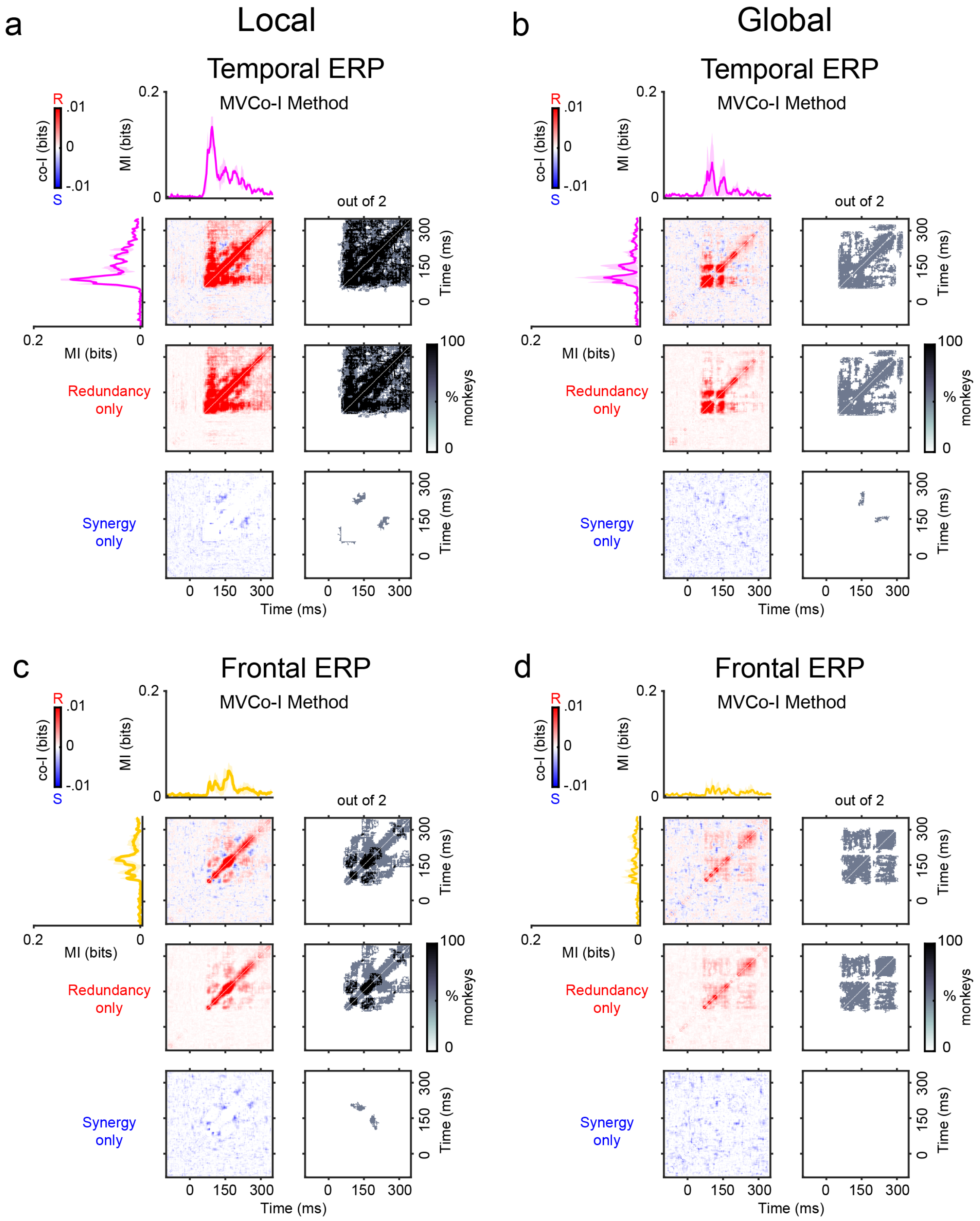

Figure S15: Temporal synergy and redundancy within ERP signals in the auditory and frontal electrodes using the MVCo-I Method (Experiment 2: Local/Global Task). MVCo-I revealed synergistic and redundant temporal patterns within auditory ERP signals in the Local (Panel **a**) and Global (Panel **b**) contrasts; and within frontal ERP signals in the Local (Panel **c**) and Global (Panel **d**) contrasts. MI (solid traces) between standard and deviant trials for auditory (pink color) and frontal (orange color) responses averaged across the two monkeys. The corresponding electrodes used for the MVCo-I method are depicted in Fig. 1b. Error bars represent standard error of the mean (S.E.M). Temporal co-I was computed within the corresponding signal (ERP) across time points between -100 to 350 ms after tone presentation. The average of the corresponding electrodes across monkeys is shown for the complete co-I chart (red and blue plots); for positive co-I values (redundancy only; red panel); and negative co-I values (synergy only; blue plot).

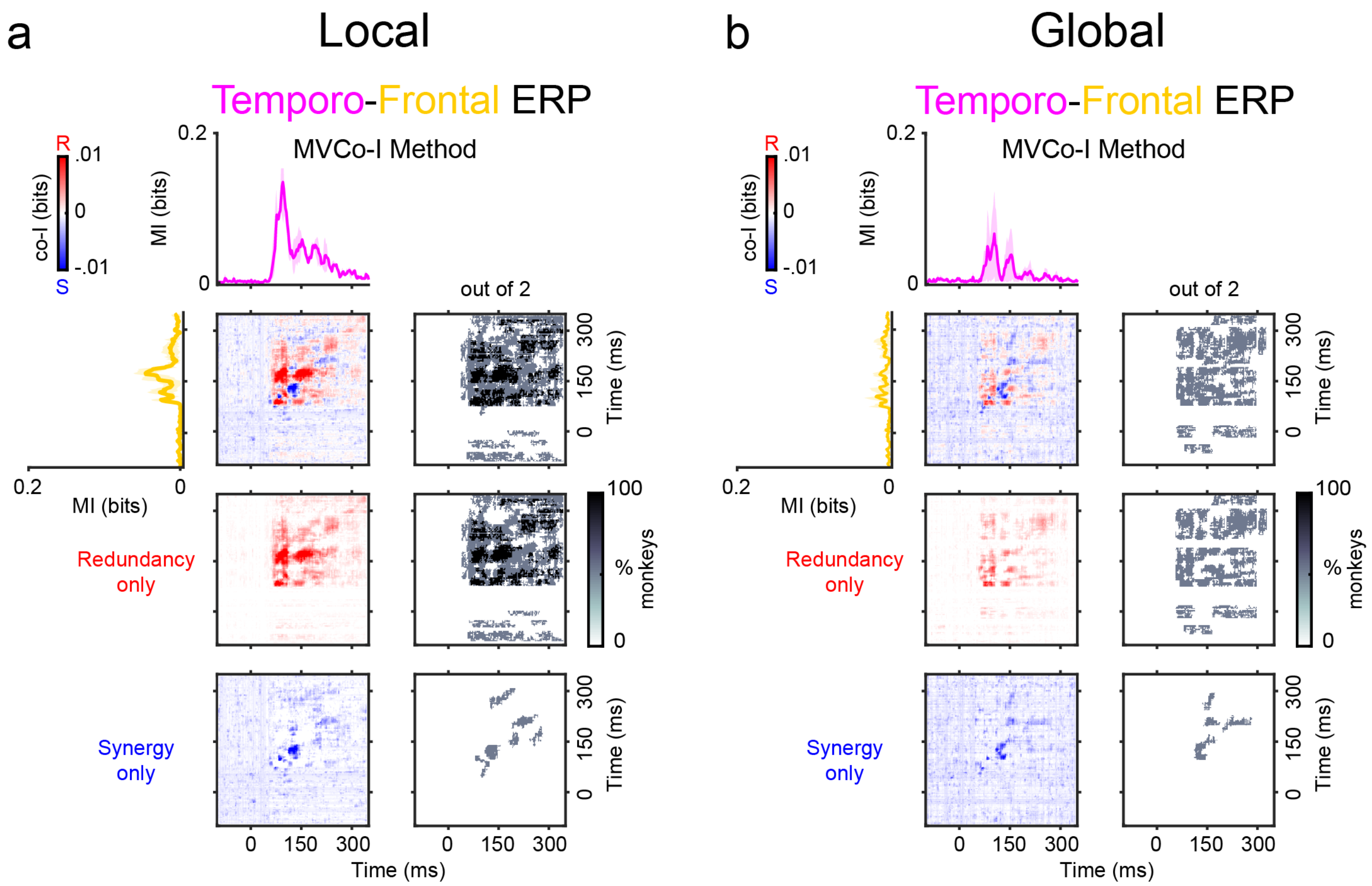

Figure S16: Spatio-temporal synergy and redundancy between temporal and frontal ERP signals using the MVCo-I Method (Experiment 2: Local/Global Task). MVCo-I revealed synergistic and redundant temporal patterns between auditory and frontal ERP signals in the Local (Panel **a**) and Global (Panel **b**) contrasts. MI (solid traces) between standard and deviant trials for auditory (pink color) and frontal (orange color) responses averaged across the two monkeys. The corresponding electrodes used for the MVCo-I method are depicted in Fig. 1b. Error bars represent standard error of the mean (S.E.M). Temporal co-I was computed within the corresponding signal (ERP) across time points between -100 to 350 ms after tone presentation. The average of the corresponding electrodes across monkeys is shown for the complete co-I chart (red and blue plots); for positive co-I values (redundancy only; red panel); and negative co-I values (synergy only; blue plot).

Supplementary Methods

Details of the neurocomputational model

Microstructure

Each area consists of two neuronal layers, each of 625 (25x25) cells, one containing excitatory cells and one containing inhibitory ones (in what follows, referred to as e- and i-cells, respectively). To avoid any potential edge effects, layers have a toroidal structure: the top edge is adjacent to the bottom one, and the left edge is adjacent to the right one. In line with Wilson-Cowan models (1), a single pair of e- and i-cell models the average activity of a local population of pyramidal neurons and underlying inhibitory interneurons within one cortical column (grey matter under approximately 0.25 square mm of the cortical surface). Cells are modelled as graded-response neurons (see below).

Each e-cell is restricted to send projections to the 19x19 e-cell neighbourhood within the same area, to topographically corresponding 19x19 e-cell patches in connected areas, and to a 5x5 i-cell patch in the inhibitory layer of the same area (Fig. 1E). The probability of a synapse to be created between an e-cell and another cell falls off with their distance (2) according to a Gaussian function clipped to 0 outside the relevant neighbourhood. This produces a sparse, patchy and topographic connectivity, as typically found in the mammalian cortex (3; 4).

Membrane dynamics

The state of an (excitatory or inhibitory) cell e at time t is uniquely defined by its membrane potential $V(e,t)$, determined by the following equation:

$$\tau \frac{dV(e,t)}{dt} = -V(e,t) + k_1(V_{in}(e,t) + k_2\eta(e,t)) \qquad (1)$$

where $V_{in}(e,t)$ is the sum of all postsynaptic inputs acting upon cell e (see Eq. (2)), $\eta(e,t)$ is a white noise process with uniform distribution over [–0.5, 0.5], τ is the cell's membrane time constant (note that e- and i-cells have different τ, see Table S1), and k_1 and k_2 are scaling constants. Note that the activity of each e-cell is intrinsically noisy, simulating the spontaneous baseline firing of real neurons (i-cells have k_2=0). The total input to a cell e is defined as:

$$V_{in}(e,t) = (\Sigma \text{ E/IPSPs}) - k_G\ \omega_G(e,t) \qquad (2)$$

where ΣE/IPSPs is the sum of all excitatory and inhibitory postsynaptic potentials – I/EPSPs; inhibitory synapses are given a negative sign – acting upon neural cluster (cell) e at time t,

$\omega_G(e,t)$ is the global (or area-specific) inhibition (see Eq. (3)) and k_G is a scaling constant. Note that each e-cell gets exactly one IPSP from its twin i-cell (see Fig. 1E).

The global inhibition mechanism is an area-specific inhibitory loop that prevents overall network activity from falling into non-physiological states (5). (Note that k_G =0 for i-cells: for simplicity, global inhibition acts only on e-cells.) The global inhibition $V_G(A,t)$ for model area A at time t is defined by:

$$\tau_G \frac{dV_G(A,t)}{dt} = -V_G(A,t) + \sum_{e \in A} O(e,t) \qquad (3)$$

where $\sum_{e \in A} O(e,t)$ is the sum of all e-cell outputs within area A (see Eq. (4)) and τ_G is the global inhibitory response time constant.

All cells produce a graded response representing the average firing rate of the neural cluster; in particular, the output (transformation function) of an e-cell *e* at time *t* is defined as:

$$\mathrm{O}(e,t) = \begin{cases} 0 & \mathit{if}\ V(e,t) \leq \varphi(e,t) \\ V(e,t) - \varphi(e,t) & \mathit{if}\ 0 < (V(e,t) - \varphi(e,t)) \leq 1 \\ 1 & \mathit{otherwise} \end{cases} \qquad (4)$$

Eq. (4) above is a piecewise-linear sigmoid function of the e-cell's membrane potential $V(e,t)$, clipped into the range [0, 1] and with slope 1 between the lower and upper thresholds ($\varphi(e,t)$, $\varphi(e,t)$+1). The output $O(i,t)$ of an i-cell i is 0 if $V(i,t)$ < 0, and $V(i,t)$ otherwise (i.e., unlike e-cells, i-cells do not saturate, reflecting that real interneurons show little firing rate adaptation).

The threshold $\varphi(e,t)$ of an e-cell is not constant but depends on the cell's recent activity, so that the more active the cell, the higher the threshold (see Eq. (5)). This implements a simple form of homeostatic adaptation, or neuronal fatigue (6):

$$\varphi(e,t) = \alpha\, \omega(e,t) \qquad (5)$$

where $\omega(e,t)$ is the estimated time-average of cell *e*'s recent output (see Eq. (6)) and α is a scaling constant (adaptation strength). The estimated time-average $\omega(e,t)$ of a cell's output is computed by numerically integrating Eq. (6) below with time constant τ_A, assuming $\omega(e,t)$=0 at time t=0:

$$\tau_A \frac{d\omega(e,t)}{dt} = -\omega(e,t) + O(e,t) \qquad (6)$$

Table S1 *Model parameters*

τ_e = 2.5	e-cells time constant (time steps) – Eq. (1)
τ_i = 5	i-cells time constant (time steps) – Eq. (1)
k_1 = 0.01	Input scaling constant – Eq. (1)
$k_2 = 150\sqrt{(24/\Delta t)}$	Noise amplitude (Δt = 0.1 ms) – Eq. (1)
$\eta \sim U[-0.5, 0.5]$	Noise distribution – Eq. (1)
τ_G = 60	Global inhibition time constant – Eq. (3)
k_G = 95	Global inhibition strength – Eq. (2)
α = 100	Adaptation strength – Eq. (5)
τ_A = 50	e-cells' time-averaging time constant – Eq. (6)

REFERENCES

[1] Wilson, H. R. & Cowan, J. D. A mathematical theory of the functional dynamics of cortical and thalamic nervous tissue. *Kybernetik* **13**, 55–80 (1973).

[2] Braitenberg, V. & Schüz, A. *Cortex: Statistics and Geometry of Neuronal Connectivity* (Springer, Berlin, Heidelberg, 1998).

[3] Amir, Y., Harel, M. & Malach, R. Cortical hierarchy reflected in the organization of intrinsic connections in macaque monkey visual cortex. The *Journal of Comparative Neurology.* **334**, 19–46 (1993).

[4] Kaas, J. H. Topographic maps are fundamental to sensory processing. *Brain Research Bulletin.* **44**, 107–112 (1997).

[5] Braitenberg, V. Cell assemblies in the cerebral cortex. In Heim, R. & Palm, G. (eds.) *Theoretical Approaches to Complex Systems*, Lecture Notes in Biomathematics, Vol. 21, 171–188 (Springer Verlag, Berlin, 1978).

[6] Matthews, G. G. *Neurobiology: Molecules, Cells and Systems* (Wiley-Blackwell, 2001)
